# Effects of a phonological awareness program on English reading and spelling among Hong Kong Chinese ESL children

**DOI:** 10.1007/s11145-012-9383-6

**Published:** 2012-05-22

**Authors:** Susanna S. S. Yeung, Linda S. Siegel, Carol K. K. Chan

**Affiliations:** 1 Department of Psychological Studies, The Hong Kong Institute of Education, Tai Po, Hong Kong SAR; 2 Faculty of Education, The University of Hong Kong, Pok Fu Lam, Hong Kong; 3 Department of Educational and Counselling Psychology, and Special Education, The University of British Columbia, Vancouver, BC Canada

**Keywords:** English reading, Biliteracy, Chinese ESL children, Phonological awareness instruction

## Abstract

This study investigated the effects of a 12-week language-enriched phonological awareness instruction on 76 Hong Kong young children who were learning English as a second language. The children were assigned randomly to receive the instruction on phonological awareness skills embedded in vocabulary learning activities or comparison instruction which consisted of vocabulary learning and writing tasks but no direct instruction in phonological awareness skills. They were tested on receptive and expressive vocabulary, phonological awareness at the syllable, rhyme and phoneme levels, reading, and spelling in English before and after the program implementation. The results indicated that children who received the phonological awareness instruction performed significantly better than the comparison group on English word reading, spelling, phonological awareness at all levels and expressive vocabulary on the posttest when age, general intelligence and the pretest scores were controlled statistically. The findings suggest that phonological awareness instruction embedded in vocabulary learning activities might be beneficial to kindergarteners learning English as a second language.

## Introduction

Phonological awareness, the conscious ability to identify and manipulate speech sounds mentally, is a very important literacy skill that children have to acquire in early years (e.g., Whitehurst & Lonigan, 1998). It has been demonstrated repeatedly as a good predictor of later reading outcomes (Blachman, 1997; Muter & Snowling, 1998; Wagner, Torgesen, & Rashotte, 1994). Similarly, children who lag behind in developing phonological awareness skills are likely to be at-risk for reading difficulties (Bradley & Bryant, 1983; Torgesen, Wagner, & Rashotte, 1997). Do these findings for monolingual English speakers generalize to ESL children who have a first language (L1) significantly different from English? It is important to explore the answers to this question because underdeveloped English language and literacy skills in ESL learners may undermine school achievement (August & Hakuta, 1997).

A large volume of research has demonstrated the beneficial effects of phonological awareness instruction on reading for English native speaking children (e.g., Bus & van IJzendoorn, 1999; Ehri et al., 2001; Snow, Burns, & Griffin, 1999). Recently, research has shown that phonological awareness instruction promotes growth in reading among young children from diverse linguistic backgrounds with English as a second language (L2) (Ganschow & Sparks, 1995; Lesaux & Siegel, 2003). However, there are few studies that have examined the effects of phonological awareness instruction on Chinese ESL children in a non-English speaking environment such as Hong Kong who generally have very little exposure to phonological instruction in their early years (Hanley, Tseng, & Huang, 1999; Holm & Dodd, 1996; Huang & Hanley, 1995). The aim of this study was to examine whether phonological awareness instruction helps to promote phonological awareness and reading skills of kindergarten Chinese ESL learners.

In this study, an experiment was conducted to examine young Chinese ESL children’s gains in phonological awareness, oral language proficiency, reading, and spelling as a result of language-enriched phonological awareness instruction that integrated oral language and phonological awareness learning in preschool classrooms.

Consistent with research on English reading in monolingual English speakers, phonological awareness has repeatedly been reported to be associated uniquely with the L2 reading development of ESL learners with different native languages (e.g., Chiappe, Siegel, & Wade-Woolley, 2002; Gottardo, Yan, Siegel, & Wade-Woolley, 2001; Lesaux & Siegel, 2003). Research on ESL children from English-speaking environments generally suggests that ESL and English speaking children show comparable performances in word decoding and other component skills 1 or 2 years after they enter elementary schools (e.g. Chiappe et al., 2002; Lesaux & Siegel, 2003). Generalizing findings from these studies to Chinese ESL children in a non-English speaking environment such as Hong Kong remains doubtful for a few reasons. First, Hong Kong children learn English as a school subject but their exposure to the English language is generally limited. It has been shown that inadequate L2 speech input may have a negative impact on L2 reading development (Meador, Flege, & MacKay, 2000). Second, the instructional practices in the Chinese speaking environment emphasize the “look and say” method (Holm & Dodd, 1996). Learning to read Chinese and English thus relies heavily on rote memory. Consequently, children may not pay attention to letter sounds or letter names within a word in the word reading process. Third, the phonological awareness skills, both in Chinese and English, of Chinese ESL learners are poorer than those of English native speakers (McBride-Change, Bialystok, Chong, & Li, 2004). This may be due to the instructional experiences as described above or to the characteristics of the Chinese language, which has virtually no consonant clusters. It has been suggested that experiences with syllables having complex phonemic structures prime the development of phonological awareness even before the child learns to read (Cheung, Chen, Yip-Lai, Wong, & Hill, 2001). Previous research has shown that Hong Kong ESL children are relatively weak in phonological awareness in both languages compared to other Chinese reading groups such as Chinese children from Mainland China and Canada because there is no phonetic code to aid Chinese reading for Hong Kong children (McBride-Chang et al., 2004).

Despite such a linguistic and educational environment that does not encourage Hong Kong children to develop phonological awareness skills, recent investigations have demonstrated the importance of phonological awareness in the early L2 reading development of Chinese ESL learners (Cheung et al., 2010; Keung & Ho, 2009; McBride-Chang & Ho, 2005; McBride-Chang & Kail, 2002; McBride-Chang & Treiman, 2003). For example, McBride-Chang and Kail (2002) examined the role of phonological awareness at the syllable level, speeded naming, visual processing and speed of processing in beginning English reading performance of kindergarten students in Hong Kong. Phonological awareness was reported to be the strongest predictor of reading among various measures. Other levels of phonological manipulations (at rhyme and phoneme levels) have been shown to explain a significant amount of variance in Hong Kong students’ English word reading in junior primary grades after controlling for age and intelligence (Keung & Ho, 2009). Research evidence has supported the role of phonological awareness in different forms in facilitating the early L2 reading of Hong Kong children.

In most of the studies involving Chinese kindergarteners (e.g., McBride-Chang & Ho, 2005; McBride-Chang & Kail, 2002; McBride-Chang & Treiman, 2003), only phonological awareness tasks at the syllable level were used. It is worth noting that previous work on English reading has indicated that more fine-grained phonological awareness, such as rime and phoneme, is related more to English reading development (Adams, 1990; Bradley & Bryant, 1983). The present study attempted to address this issue by incorporating measures of phonological awareness at the levels of syllable, rime and phoneme and investigating the growth of phonological awareness at varied levels after receiving phonological awareness instruction.

The significant role of phonological awareness in L2 reading development has prompted researchers to examine the effects of phonological awareness instruction in helping ESL children to acquire L2 reading skills. For instance, Ganschow and Sparks (1995) demonstrated that explicit and systematic training in Spanish phonological awareness was able to improve English phonological awareness, word reading and spelling in Spanish–English bilingual children who may have had reading difficulties. Lesaux and Siegel (2003) demonstrated that ESL children from diverse linguistic backgrounds performed as well as L1 speakers in reading skills after receiving 1 year of phonological awareness instruction in kindergarten. The findings support the idea that early theory-driven instruction is effective in helping ESL learners who have little or no prior input of English oral language to acquire English reading skills. Nevertheless, as aforementioned, the generalizability of these results to Chinese ESL children remains to be investigated. Moreover, relatively few studies in the field have involved class teachers in undertaking instruction and working with children in large groups in a kindergarten setting.

Many ESL children, at the time they start to learn to read a L2, have limited oral language proficiency (August & Hakuta, 1997). In Hong Kong kindergarten classrooms, L2 oral inputs are generally limited and both reading and vocabulary acquisition tend to rely more on print than on speech (Cheung et al., 2010). As there is a lack of English exposure outside the classroom, Hong Kong young children tend to have weak oral language proficiency.

According to the lexical restructuring hypothesis (Metsala & Walley, 1998), when children’s vocabularies expand rapidly and spoken words become more and more similar phonetically (e.g. hot and pot are different by a single phoneme), they must begin to represent the words with “segmental phonology”. The segmental representation of word phonology increases at fine-grained level as development continues. The degree to which the restructured segmental representation has taken place contributes to the development of phonological awareness and subsequently influences the processes of learning to read and write. Based on this model, vocabulary size is related to one’s phonological awareness and, in turn, contributes to reading performance. On the other hand, the acquisition of literacy may influence the further development of phonological awareness and thus may lead to re-organization of the segmental representation or lexical categories. In this vein, phonological awareness may contribute to the further development of lexicon. The lexical restructuring hypothesis contends that there are close relationships between vocabulary growth and phonological awareness and reading development. Enhanced phonological awareness stimulated vocabulary growth. We argue that the lexical restructuring hypothesis is applicable to the ESL children in Hong Kong because vocabulary is the main focus in Hong Kong kindergarten English curriculum and children are expected acquire basic vocabulary during the kindergarten years. Therefore, despite weak oral language proficiency, their lexicon undergoes restructuring as a result of vocabulary growth.

Previous studies of English speaking children and ESL children learning alphabetic L1 have shown that oral language proficiency is associated with reading skills (Bernhardt & Kamil, 1995; Gottardo, Collins, Baciu, & Gebotys, 2008; Lindsey, Manis, & Bailey, 2003; Nation & Snowling, 2004). In this connection, in designing instructional program for ESL children, the level of oral language proficiency, particularly vocabulary, has to be taken into consideration. Some research evidence has supported this idea. For example, Ayres (1998) integrated a phonological approach with literacy-based activities such as word games and storytelling. It was found that children benefited most when they were exposed to literacy-based activities first and then to direct and explicit instruction about phonological awareness. Literacy building activities seem to facilitate the explicit instruction in segmentation of words. Therefore, the current study was designed to test the effects of phonological awareness instruction integrated with vocabulary learning activities.

In summary, previous studies have established a relationship between the L2 phonological awareness and L2 reading skills of Chinese ESL children. This study was designed to examine the effects of language-enriched phonological awareness instruction on phonological awareness skills, oral language proficiency, reading and spelling in young Chinese ESL children from Hong Kong. There were two conditions: (a) language-enriched phonological awareness instruction and (b) English language activities focusing on vocabulary learning and word writing as a treated comparison. Specifically, the following research questions were addressed:

1. Would children with language-enriched phonological awareness instruction perform better at posttest than the comparison group children on (a) phonological awareness skills at syllable, rhyme and phoneme levels, (b) receptive and expressive vocabulary, and (c) word reading and pseudoword reading, and (d) spelling?
2. What oral language and phonological awareness measures would predict children’s improvements on word reading and spelling for the instructional and comparison groups?

## Method

### Participants

At the beginning of the study, four kindergartens located in Hong Kong participated. Seventy-six children from three kindergartens completed the instructional program and the posttest. There were 38 children in the instructional group and 38 in the comparison group. These children did not differ significantly from the dropouts with respect to age or all pretest measures, *p*s > 0.10. The class teachers reported that no participating children were observed to show any signs of developmental disabilities or neurological disorders.

All the participating schools are typical Hong Kong kindergartens in which Cantonese is used as the medium of instruction and English is regarded as a school subject. Children in these schools have 2–3 English lessons per week, each of approximately 20–30 min, with Native English Teachers (NETs). They also do English and Chinese writing (copying) every day, instructed by the class teachers and the time spent on this activity is approximately 20–30 min per day. The teachers of the participating kindergartens reported that no systematic phonics teaching was provided.

All children in this study spoke Cantonese at home. The parents of these children are mainly lower class or lower-middle class Hong Kong people who would not communicate with their child in English at home. Generally, the children were only exposed to English oral language at school in the English lessons delivered by the NETs.

All children were tested and participated in the instructional program in their first semester of the school year. There were 38 boys and 38 girls who completed the study and their mean age was 5.14 (SD = 0.23; range = 4 years, 9 months to 5 years, 9 months).

### Teachers’ background

There were six participating teachers, three from each of the instructional and comparison groups, all of whom possessed a higher diploma in early childhood education from Hong Kong tertiary institutions. The average teaching experiences of the instructional and comparison group teachers were 4 and 4.5 years respectively. All of the teachers were Cantonese speakers.

### Language-enriched phonological awareness instruction

The current instruction was supplementary to existing English language and reading instruction that the children received in their schools. Information about the English curriculum from each school was collected and there were no differences among the three participating schools in terms of the learning goals and content.

The current instructional program was based on the work by Bennett and Ottley (2000). Their program followed a fixed sequence: awareness of sound as a unit of words, syllable segmentation, rhyming, onset and rime and discrimination. In this study, the children went through only the first five instructional components of the program. They were taught to understand that one English word may have multiple syllables and to tap syllables within a word. Rhyming skills and phoneme identity (initial and final) were also taught. In all the learning activities, a lot of pictures were used to sustain the children’s interest and attention and no print was used. The instruction used for this study, therefore, was mainly auditory training to promote the participants’ awareness of the sound structures within English words. Existing English instruction in Hong Kong does not use explicit teaching of phonological concepts and skills.

In each stage, practice time was included. In each learning activity, clear learning outcomes and necessary resources were stated. The program was activity-oriented and paper-and-pencil tasks were not included. For example, in learning syllable awareness, the children were asked to tap syllables on body parts and practices were done with orally presented words with different numbers of syllables (e.g., four for watermelon). Based on the work of Bennett and Ottley (2000), the researchers made changes in the English vocabulary used to match the participants’ oral language proficiency. For example, the word “rake”, which is uncommon for young children in Hong Kong, was replaced by the word “cake” in one of the rhyming sessions.

In each session, the children were first taught the vocabulary to be used for the day. The vocabulary instruction was direct and explicit. Children were first provided with kid-friendly definition of the targeted words and then exposed to the vocabulary in meaningful contexts and simple English sentences. They were then instructed to play games that provided them with rich opportunities to name the pictures associated with the targeted words. There was some overlapping (30 %) between the targeted vocabulary and the vocabulary tests used in this study. However, the vocabulary was chosen based on the content of the phonological awareness part. This was then followed by explicit instruction in a specific phonological awareness skill. For instance, after storytelling and teaching the vocabulary of *king* and *ring*, the teacher would incorporate them in phonological awareness instruction. The teacher would hold up three picture cards depicting *king*, *ring* and *man* and ask which two rhymed with each other. The teacher would model the phonological awareness skills before each game began and the children were given ample opportunities to participate and respond. The learnt vocabulary and phonological awareness skills were reviewed in each lesson. The teachers were provided with all necessary teaching materials to ensure consistency between groups in different schools.

### The comparison group

Teachers were given the targeted word list of the language-enriched phonological awareness instruction and were asked to teach the words for the comparison group children using word instruction and copying activities. Some of the targeted words overlapped with their existing English curriculum. The comparison instruction was similar to the typical Hong Kong kindergarten English instruction. Similar to the language-enriched phonological awareness instruction, the children went through 24 30-min sessions over a period of 12 weeks. No phonological awareness instruction was provided. The two instructions were comparable in terms of learning time and the targeted vocabulary.

### Pretest and posttest assessments

All measures were administered individually with instructions in Cantonese, which is the participants’ spoken language, by trained experimenters.^1^ The English items were administered in English. These measures are described in detail below.

#### Nonverbal intelligence

Raven’s Colored Progressive Matrices (Raven, Court, & Raven, 1976), which is a measure of non-verbal reasoning, was adopted for estimating the participants’ non-verbal intelligence. As the participants were preschoolers, the short form was used. In each item, a visual matrix with one missing part was presented and the children were asked to select, from six alternatives, the one that best completed the matrix. The first set of the items from the short form was used and there was a total of 24 items. One mark was given for each correct response, and the maximum score was 24. The internal consistency reliability coefficient was 0.72.

#### English reading and spelling

##### English word reading

The test previously used by McBride-Chang and Kail (2002) was used as a measure of English word recognition. This consists of 30 common English words from common English textbooks for Hong Kong preschoolers. The participants were instructed to pronounce the printed words one by one. One point was given for each correct pronunciation and the maximum score was 30. The internal consistency reliability coefficient was 0.94.

##### English spelling

In this developmental spelling test, there were a total of 6 simple and unfamiliar English words in CVC or CCVC structure (*fan*, *top*, *rug*, *pin*, *fist*, *glad*). The experimenter pronounced the words in isolation first and then read a simple English sentence with the target word. The target word was then repeated. The participants were asked to write the word in order to practise spelling it. If a child did not know the word, he or she was encouraged to guess the spelling from the pronunciation. It was of interest to assess emergent spelling in English and examine the children’s knowledge of phonological elements in the words. Scoring was based on the number of correct phonemes contained in the spelled words. For example, for the word *rug*, there are three phonemes and a maximum of 3 points were given if the child spelled this word correctly. The maximum score for this test was 20. The reliability of this test could not be calculated because the scale had zero variance items in the pretest.

##### Pseudoword reading

The pseudoword reading test consisted of 10 non-words in VC, CVC and CVCC structures (af, *pem*, *fip*, *sep*, *gan*, *bim*, *tay*, *pesh*, *hafe*, and *vist*). The children were asked to read the words aloud. The scoring was based on the number of correct phoneme pronounced. The maximum score was 30. Again, the internal consistency reliability could not be determined because there were zero variance items.

#### Oral language proficiency

##### English receptive vocabulary

The Peabody Picture Vocabulary Test-Revised (PPVT) Form M (Dunn & Dunn, 1981) was used to measure English receptive vocabulary. The English words were presented orally by the experimenter and the children were asked to point to the one of four pictures that was associated with the given word. Twenty-four-items from the 2–6 year-old subset were administered. One point was given to correct identification and the maximum score was 24. The internal consistency reliability was 0.66.

##### English expressive vocabulary

A picture naming task was adopted to assess the children’s expressive vocabulary. Each child was presented with 15 pictures of common objects such as a frog, a tree and so on. The children were prompted to name these one by one. One mark was given to each correct naming and the maximum score was 15. The internal consistency reliability was 0.83.

#### Phonological awareness

##### Syllable deletion (Learning Disabilities Association of Alberta, 2009)

This was a phonological awareness task in which the children were asked to delete a syllable from a 3-syllable compound word or phrase (e.g. say *Butterfly* without *fly*). There were 2 practice items and 8 test items. One point was given for a correct deletion and the maximum score was 8. The internal consistency reliability was 0.67.

##### Rhyme detection (Muter, Hulme, Snowling, & Taylor, 1997)

This task consisted of 10 items. A stimulus word was presented to the children and then they were asked to choose the word that rhymed with it or had the same ending sounds from a list of three words (e.g., “Which word rhymes with the word “boat”: foot, bike or coat?”). Pictures of the words were shown when the experimenter gave the instruction. One point was given for each correct response. There were two demonstration items done before the actual testing. The maximum score was 10. The internal consistency reliability was 0.60.

##### Rhyme generation (Learning Disabilities Association of Alberta, 2009)

This test was the second task to assess the children’s rhyme awareness. One practice item and 8 test items were administered. The children were asked to provide a word or nonword that rhymed with the word provided (*pear*, *dish*, *cap*, *ring*, *rock*, *snake*, *rice* and *bed*). One mark was given for any possible correct answer and the maximum score was 8. There were some zero variance items and, therefore, the reliability could not be calculated.

##### Phoneme identification (Muter et al., 1997)

This task was used to assess the children’s phonemic sensitivity. In this 8-item task (*horse*, *fish*, *knife*, *gate*, *dog*, *ship*, *card* and *bone*), the children were shown a series of pictures. The experimenter pronounced the word once and then the first part of the word (2 phonemes) associated with the picture. The children were then asked to finish the word by providing the last phoneme. The number of phonemes pronounced correctly by the children was recorded. For example, for the word “dog”, the experimenter would pronounce the word once and then provided the first two phonemes (“do”). The correct response was */g/*. Before the test items, two demonstration items were provided. The maximum score was 8. The internal consistency reliability was 0.60.

##### Initial phoneme deletion (Learning Disabilities Association of Alberta, 2009)

This test was used to assess the children’s phoneme awareness. There were 2 practice trials and 6 test trials. All items were one-syllable words in CVC, CCVC or CCVCC structures (*pig*, *fix*, *car*, *mat*, *rice*, *sport*). It was expected that the children might be unfamiliar with these words. The experimenter read out each word aloud and asked the children to say the target word without the initial phoneme. One point was given for each correct response and the maximum score was 6. The internal consistency reliability could not be determined for zero variance items.

#### Letter knowledge

##### Letter identification

The children were presented with English letters, all lower case, in random order and asked to say their names. The score was based on their numbers of correct namings. The maximum score for this test was 26. The internal consistency reliability was 0.90.

### Procedures

The school principals were approached and their agreement to participate in the study was sought. Only children with parental consent joined the instructional program. The pretest and posttest were conducted in a quiet room in each school.

Prior to the commencement of the instructional program, teacher training was provided by the researcher (the first author) to all the participating teachers. The researcher explained the rationale of the training, program structure, learning objectives, and characteristics of the activities to the teachers. To help the teachers to be equipped better with the linguistic skills required for running the sessions, the researcher also provided a training session in English phonology, phonetics and phonological awareness. All teachers were encouraged to deliver the activities in English. However, from the lesson observations, some Cantonese (the spoken language of Hong Kong Chinese) was used to help the children to understand the instructions. During the program implementation, the researcher had weekly meetings with the teachers to discuss the children’s progress, clarify the learning outcomes and receive comments from teachers about the program. Altogether 12 meetings were conducted in each school. Contingent feedback was provided when needed during meetings on instructional pacing and teaching methods and techniques. All teachers were blind to the assessments conducted in this study.

Following the completion of the pretest, the children were assigned randomly to the instruction group (*n* = 38) and the comparison group (*n* = 38). Children in both conditions were taught in groups of 12–13. There were 2 lessons per week for a total of 12 weeks. Each lesson lasted for 30 min.

Fidelity checks were conducted to examine the program adherence and differentiation. For each school, 12 lessons for each group (50 % of the total number of lessons) were observed for instructional adherence and differentiation. During the lesson observation, the researcher rated the following observations on a self-developed checklist: (1) All key components of the lesson have been covered; (2) Teacher has used the teaching materials in general accordance with the lesson plan; (3) There have been no major distraction and/or disruptions during the lesson; (4) Teacher has attempted to engage children’s attention in the lesson.

## Results

The demographic characteristics of the participants are reported in Table 1. Table 2 shows the means for all tests administered at pretest and posttest in each group. Preliminary analyses with analysis of variance (ANOVA) indicated that the two groups did not differ in demographics (gender, age and parent education) with *p*s > 0.70. Also, there was no significant difference in all pretest scores between two groups as revealed by the ANOVAs, *p*s > 0.65.

**Table 1:** Demographic characteristics of the participants by groups

	Instructional group			Comparison group		
	*M*	SD	Range	*M*	SD	Range
Age	5.12	0.26	4.75–5.75	5.12	0.19	4.75–5.75
General intelligence (Raven)	13.42	2.49	4–21	13.71	3.22	5–22
Parent education	1.98	0.57	1–3	2.06	0.68	1–3

Parent education was derived by the maternal educational levels with the following codes: 1 = primary, 2 = secondary, 3 = college, 4 = postgraduate

**Table 2:** Means and SD on all measures by groups

	Instructional group		Comparison group		*F*(1, 75)
	*M*	SD	*M*	SD	
Reading and spelling					
Word reading					
t1	6.39	5.68	6.79	6.94	
t2	9.53	7.47	7.74	7.82	8.41**
Pseudoword reading					
t1	0.84	1.31	1.37	3.02	
t2	2.84	4.42	1.34	4.20	5.95^+^
Spelling					
t1	0.34	1.44	0.37	1.58	
t2	3.32	3.98	1.51	2.36	7.40**
Oral language proficiency					
PPVT					
t1	11.66	3.27	11.79	3.81	
t2	12.68	3.08	12.59	3.30	0.13, NS
Picture naming					
t1	6.13	3.35	5.68	3.45	
t2	8.45	3.45	6.84	3.87	6.89**
Phonological awareness					
Syllable deletion					
t1	4.24	1.99	3.97	1.60	
t2	5.47	1.59	4.29	2.08	9.66***
Rhyme detection					
t1	4.08	2.06	3.66	2.20	
t2	5.55	1.80	4.08	1.78	15.07***
Rhyme generation					
t1	0.16	0.44	0.08	0.27	
t2	1.55	1.13	0.24	0.85	34.49***
Phoneme identification					
t1	3.42	1.54	3.16	1.97	
t2	4.87	1.56	3.50	1.86	16.67***
Phoneme deletion					
t1	0.45	0.50	0.42	0.50	
t2	0.97	1.17	0.32	0.87	9.28***
Letter knowledge					
Letter identification					
t1	21.37	5.15	20.50	6.25	
t2	23.05	4.34	22.55	4.23	0.009, NS

*t1* pretest, *t2* posttest, *NS* non-significant, ^+^ marginal significance; ** *p* < 0.01; *** *p* < 0.001

A fidelity check indicated that 97 % of the lessons observed for the instructional group were recorded as following the assigned lesson plans and teaching the key lesson components as stated. In 97 % of the lessons teaching materials were used according to the lesson plans. In all the lessons observed, the teachers attempted to engage the children’s attention. No phonological instruction was provided for children in the comparison group. The results indicated that the current implementation had good fidelity, indicating a high level of program differentiation between the two conditions and program adherence.

### Instructional effects on phonological awareness, oral language proficiency, reading and spelling

The instructional effects were examined by performing separate Analysis of Covariance (ANCOVA) tests on measures of reading and spelling, with group as a between-subject factor and pretest scores, age, and general intelligence (Raven) as covariates. Multivariate analysis of covariance (MANCOVA) was conducted on measures of oral language proficiency and phonological awareness as measures within the same construct tended to correlate with one another.

#### Phonological awareness

Multivariate analysis of covariance was conducted with group membership as the independent variable and the six phonological awareness measures employed in this study (syllable deletion, rhyme detection, rhyme generation, phoneme identification, phoneme deletion and pseudoword reading) as dependent variables. Age, general intelligence and the pretest scores of the measures were entered as covariates. The results from MANCOVA revealed that there was a main effect of group, Wilks’ Lambda = 0.57, *F*(6, 70) = 6.65, *p* < 0.001, partial η^2^ = 0.42, indicating that, as expected, the instructional group had significantly higher phonological awareness scores than the comparison group. No main effect was found for age, Wilks’ Lambda = 0.89, *F*(6, 70) = 1.28, *p* = 0.28, and general intelligence, Wilks’ Lambda = 0.86, *F*(6, 70) = 1.67, *p* = 0.14. To avoid the inflation caused by a Type I error, we adopted an adjusted alpha level based on Bonferroni correction. Follow-up ANCOVA analyses with adjusted alpha value (0.05/6 = 0.008) indicated that there were significant effects of group on all phonological awareness measures except for the pseudoword reading measure. The results showed that children in the instructional group scored significantly higher than those in the comparison group on syllable deletion, *F*(1, 75) = 9.66, *p* < 0.001, Cohen’s *d* = 0.64; rhyme detection, *F*(1, 75) = 15.07, *p* < 0.001, Cohen’s *d* = 0.82; rhyme generation, *F*(1, 75) = 34.49, *p* < 0.001, Cohen’s *d* = 1.31; phoneme identification, *F*(1, 75) = 16.67, *p* < 0.001, Cohen-*d* = 0.80; and phoneme deletion, *F*(1, 75) = 9.28, *p* < 0.001, Cohen’s *d* = 0.63. For pseudoword reading, there was only a marginally significant effect for group, *F*(1, 75) = 5.95, *p* = 0.014, Cohen’s *d* = 0.35. Taken together, the instructional program was effective in enhancing phonological awareness skills at various levels, including syllable, rhyme and phoneme levels.

#### Oral language proficiency

A MANCOVA analysis was conducted to examine the differences between the two groups on oral language proficiency skills in the posttest. PPVT (receptive vocabulary) and picture naming (expressive vocabulary) scores were entered as dependent variables, group was entered as the independent variable and age, general intelligence and pretest scores were entered as covariates. There was a main effect of the group membership which did not reach a conventional level of statistical significance, Wilks’ Lambda = 0.92, *F*(6,70) = 2.84, *p* = 0.07. Separate ANCOVAs were performed to examine the effects on receptive and expressive vocabulary with an adjusted alpha level (0.05/2 = 0.025). The results from ANCOVAs revealed that there was a main effect of group on the picture naming task, *F*(1, 75) = 6.89, *p* = 0.011, Cohen’s *d* = 0.44, with the instructional group performing better than the comparison group on expressive vocabulary in the posttest. There was, however, no main effect of group on the receptive vocabulary measure, *F*(1,75) = 0.13, *p* = 0.72. The results indicated that the current program had a specific effect on expressive vocabulary but not on receptive vocabulary.

#### Word reading and spelling

Two separate ANCOVAs were performed on English word reading and English word spelling with group as a between subject factor and age, general intelligence and pretest score as covariates. ANCOVAs revealed that there was main effect of the group on English word reading, *F*(1, 75) = 8.41, *p* < 0.01, Cohen’s *d* = 0.23, as well as English word spelling, *F*(1, 75) = 7.40, *p* < 0.01, Cohen-*d* = 0.55, indicating that the children in the instructional group performed significantly better than those in the comparison group on English reading and spelling after program implementation.

#### Letter knowledge

There was no significant main effect of group on the measure of letter identification, *F*(1, 75) = 0.009, *p* = 0.92. Letter knowledge was not the targeted area of the instructional program and the similar gains between the two groups suggested that the children acquired letter knowledge through their existing school curriculum. The experimental program did not involve print learning, demonstrating specific effects of the program on the targeted domains of literacy development.

### Predictors of word reading and spelling at Time 2

The results indicated that both oral language proficiency (expressive vocabulary) and phonological awareness at varied levels improved after the instructional program. A series of regression analyses using changes in scores from Time 1 to Time 2 was then performed to provide patterns of predictive relationships between oral language skills, phonological awareness at varied levels, reading and spelling by groups to illuminate further the role of oral language proficiency and phonological awareness on beginning reading and spelling of Chinese ESL learners. We were also interested in examining which levels of phonological awareness are important for reading and spelling.

The first set of separate hierarchical multiple regressions was performed to test whether change in phonological awareness predicted changes in English word reading and spelling uniquely after controlling for general intelligence, initial reading/spelling ability and change in oral language skills for the instructional group and the comparison group (see Table 3). In Step 1, Raven was entered in the regression analysis. Time 1 scores of reading or spelling were entered in Step 2 to control for children’s ability before the program began. Limited by the small sample size, only the measure of picture naming representing oral language skills was entered in Step 3. Phonological awareness, indexed as a composite score derived from adding the changes in raw scores of all phonological awareness measures and pseudoword reading, was entered in Step 4. For the instructional group, as shown in Table 4, changes in phonological awareness accounted for 10 %, ∆*F*(1, 37) = 4.48, ∆*R* = 0.10, *p* < 0.05, of unique additional variance of reading after controlling for general intelligence, initial reading ability and change in oral language proficiency after the instructional program. On the contrary, for the comparison group, phonological awareness did not account for additional unique variance. For spelling, differential predictive relationships also emerged between the two groups. When general intelligence and initial spelling performance were controlled statistically, changes in phonological awareness, ∆*F*(1, 37) = 11.11, ∆*R* = 0.24, *p* < 0.01, accounted for unique additional variance of change in spelling performance for children in the instructional group. Only general intelligence, ∆*F*(1, 37) = 4.48, ∆*R* = 0.11, *p* < 0.05, accounted for the unique variance of change in spelling for children in the comparison group.

**Table 3:** Final beta weight, *R* ^2^, *R* ^2^ change and *F* change for hierarchical regression equations predicting changes in word reading and spelling from general intelligence (Time 1), Time 1 performance, changes in oral language proficiency and changes in phonological awareness

	Groups							
	Instructional group				Comparison group			
	β	*R* ^2^	∆*R* ^2^	∆*F*	β	*R* ^2^	∆*R* ^2^	∆*F*
Change in English word reading								
Step 1. Raven	−0.01	0.01	0.01	0.45	−.10	0.02	0.02	0.67
Step 2. English word reading at Time 1	0.29^+^	0.12	0.11	4.47*	0.11	0.02	0.00	0.01
Step 3. Change in picture naming	0.19	0.17	0.05	2.05	0.52**	0.22	0.20	8.77**
Step 4. Change in phonological awareness^#^	0.33*	0.27	0.10	4.48*	0.20	0.25	0.03	1.41
Change in English spelling								
Step 1. Raven	0.03	0.02	0.02	0.80	0.36^+^	0.11	0.11	4.48*
Step 2. English spelling at Time 1	0.02	0.03	0.01	0.10	0.20	0.12	0.01	0.47
Step 3. Change in picture naming	0.10	0.05	0.02	0.95	0.10	0.14	0.02	0.64
Step 4. Change in phonological awareness^#^	0.51**	0.29	0.24	11.11**	0.07	0.16	0.02	0.01

^#^ A composite score derived from the sum of scores of changes in syllable deletion, rhyme detection, rhyme generation, phoneme identification, phoneme deletion and pseudoword reading

* *p* < 0.05; ** *p* < 0.01; ^+^ *p* < 0.10

**Table 4:** Final beta weight, *R* ^2^, *R* ^2^ change and *F* change for hierarchical regression equations predicting changes in English word reading and spelling from Time 1 performance and change in English phonological awareness at various levels

	Groups							
	Instructional group				Comparison group			
	β	*R* ^2^	∆*R* ^2^	∆*F*	β	*R* ^2^	∆*R* ^2^	∆*F*
Changes in English word reading								
Step 1. English word reading at Time 1	0.38*	0.12	0.12	4.78*	0.04	0.001	0.001	0.02
Step 2. Change in syllable deletion	0.22	0.16	0.04	1.75	0.43*	0.09	0.09	3.47^+^
Step 3. Change in rhyme detection	0.02	0.16	0.001	0.05	−0.27	0.17	0.08	3.30^+^
Step 4. Change in phoneme identification	0.18	0.19	0.03	1.14	0.26	0.24	0.06	2.77
English Word reading								
Step 1. English word reading at Time 1	0.35*	0.12	0.12	4.78*	0.07	0.001	0.001	0.02
Step 2. Change in syllable deletion	0.07	0.16	0.04	1.75	0.40*	0.09	0.09	3.47^+^
Step 3. Change in rhyme detection	0.005	0.16	0.001	0.05	0.30^+^	0.17	0.08	3.30^+^
Step 4. Change in phoneme deletion	0.51***	0.40	0.26	13.15***	0.07	0.18	0.004	0.17
English spelling								
Step 1. English spelling at Time 1	0.002	0.002	0.002	0.08	0.03	0.001	0.001	0.02
Step 2. Change in syllable deletion	0.07	0.003	0.001	0.03	−0.03	0.005	0.004	0.15
Step 3. Change in phoneme identification	0.16	0.05	0.05	1.68	0.38	0.08	0.08	2.87
Step 4. Change in pseudoword reading	0.67***	0.44	0.44	28.75***	−0.02	0.08	0.001	0.004
English spelling								
Step 1. English spelling at Time 1	0.003	0.002	0.002	0.08	0.20	0.001	0.001	0.02
Step 2. Change in syllable deletion	−0.04	0.003	0.001	0.03	0.03	0.005	0.004	0.15
Step 3. Change in phoneme deletion	0.40**	0.33	0.33	16.83***	0.12	0.01	0.005	0.30
Step 4. Change in pseudoword reading	0.55***	0.60	0.27	22.25***	0.15	0.02	0.01	0.31

^+^ *p* < 0.10; * *p* < 0.05; ** *p* < 0.01; *** *p* < 0.001

It was of interest in this study to examine the predictors of word reading and spelling in both groups using syllable, rhyme and phoneme measures to examine whether there was a grain size difference in accounting word reading and spelling between the two groups. Initial reading or spelling scores were entered in Step 1. For word reading, improvements in syllable deletion, rhyme detection, and phoneme identification/phoneme deletion were entered in subsequent steps because phonological awareness develops from larger grain size to smaller grain size (Anthony, Lonigan, Driscoll, Phillips, & Burgress, 2003) and Hong Kong young children tend to have weak phonemic awareness (McBride-Chang & Ho, 2005). Table 4 shows the final beta weights, *R* ^2^ change and F change in each group for English word reading and English spelling. For English spelling, we entered initial spelling score in Step 1 and improvements in syllable deletion, phoneme identification/phoneme deletion and pseudoword reading were entered in subsequent steps. We were not able to enter all the measures employed because of the sample size.

In predicting posttest English reading performance, the first regression model with phoneme identification entered in the last step showed that improvements in various forms of phonological awareness did not predict unique additional variance after controlling for initial reading ability for the instructional group. On the contrary, change in syllable awareness was a significant predictor of change in English word reading for children in the comparison group. In the second regression model with phoneme deletion entered in the final step, English phoneme deletion explained additional variance of improvement in reading, ∆*F*(1, 37) = 13.15, ∆*R* = 0.24, *p* < 0.001. Similarly, in this regression model, only syllable awareness was the significant predictor of change in English reading for the comparison group. The differential predictive relationships between two groups demonstrated a grain size effect in which different units of phonological awareness were associated with word reading of children with or without exposure to phonological awareness instruction.

For improvement in spelling, we also performed two sets of hierarchical regression analyses. In the first set of regression models, examining the effects of improvements in syllable deletion, phoneme identification and pseudoword reading, only change in pseudoword reading accounted for unique additional variance after controlling for initial spelling performance in the instructional group, ∆*F*(1, 37) = 28.75, ∆*R* = 0.44, *p* < 0.001. For the comparison group, none of the measures entered significantly accounted for changes in spelling performance. In the second set of the regression model, we entered phoneme deletion instead of phoneme identification in Step 3. The results showed that both phoneme deletion, ∆*F*(1, 37) = 16.83, ∆*R* = 0.33, *p* < 0.001, and pseudoword reading, ∆*F*(1, 37) = 22.25, ∆*R* = 0.27, *p* < 0.001, accounted for unique additional variance of improvement in English spelling for the instructional group whereas none of the phonological measures accounted significantly for English spelling in the comparison group. The pattern of predictive relationships suggested that advanced phonological awareness skills, including print-related awareness like pseudoword reading, may be involved in the spelling development of young Chinese ESL children.

## Discussion

The present study explored the effects of a 12-week language-enriched phonological awareness program on phonological awareness, oral language, word reading and spelling skills delivered by the class teachers of Hong Kong ESL kindergartners. There were three major findings. First, the instruction was found to facilitate the acquisition of phonological awareness at syllable, rhyme and phoneme levels, expressive vocabulary, word reading and word spelling to a larger extent than the comparison instruction. It should be noted that in the control condition there was very little emphasis on oral language activities but rather that the emphasis was on print learning through whole word learning and copying, the typical English language instruction in Hong Kong.

Second, changes in phonological awareness predicted improvements in word reading and spelling after controlling for the effects of general intelligence, oral language skills and the initial ability of the children participating in the current program. Last, phoneme awareness was demonstrated as the most important unit of phonological awareness in explaining beginning L2 reading of Chinese ESL children.

It should be noted that the amount of growth was the result of the instruction that lasted only for 2 sessions per week for 12 weeks. Children in the comparison group showed very little gain in various phonological awareness skills and nearly none in phoneme awareness. This suggests that the existing kindergarten reading instruction in Hong Kong, with its emphasis on exposing children to print and learning letter names, may not enable Chinese ESL children to develop sufficient phonological awareness skills to aid English word reading and spelling. It has been shown that phoneme awareness requires explicit and direct instruction among native English speaking children (Ehri et al., 2001). An important implication of the present research is that young Chinese ESL children are able to learn phonological awareness skills quickly through brief and direct instruction embedded in rich language activities that are playful and enjoyable when implemented by class teachers in preschool settings.

The current findings are generally consistent with past studies of phonological awareness instruction delivered to ESL children (e.g., Gerber et al., 2004; Leafstedt, Richards, & Gerber, 2004) to enhance beginning word reading skills. Given that this was, to the best of our knowledge, the first training study to target kindergarten-aged Chinese ESL children and that most related studies have examined intensive and small group instructional programs (e.g., Gerber et al., 2004), the findings contribute to the empirical literature that short-term phonological awareness instruction provided by class teachers is beneficial to the acquisition of L2 word reading of children learning non-alphabetic L1. Nevertheless, it has to be acknowledged that the effect size on reading and spelling was relatively small. Meta-analysis of phonological awareness instruction studies has shown that the effect size on reading is moderate in monolingual learners (Ehri et al., 2001). The beneficial effects of more intensive and comprehensive phonological awareness teaching in classroom settings need to be documented and investigated further.

We also showed that phonological awareness predicted reading and spelling after controlling for oral language skills and initial reading and spelling abilities for the instructional group but not for the comparison group. We argue that children in the comparison group had relatively weak phonological awareness and, therefore, it failed to account for individual differences in reading and spelling. The children in the instructional group acquired phonological awareness skills at varied levels and applied them to aid reading and spelling familiar and novel words.

Interestingly, our regression analyses showed that fine-grain phonological awareness (phoneme) significantly predicted the word reading and spelling for the instructional group but phonological awareness at larger grain-size (syllable) significantly explained word reading and spelling for the comparison group. The differential predictive relationships seem to suggest that the acquisition of metalinguistic skills specific to the writing system, in this case phonological awareness at rhyme or phoneme levels (Foorman, Chen, Carlson, Francis, & Fletcher, 2003; National Reading Panel, 2000; Ziegler & Goswami, 2005), is one of the necessary conditions of beginning reading development among L2 learners. Specific instruction tailored for learning specific writing system is thus needed (Luk & Bialystok, 2008). For children mainly exposed to whole word instruction, phonological awareness at the larger grain-size, as shown in our study, is involved in English reading acquisition. The findings support the idea that instructional experiences influences the course of reading development (Holm & Dodd, 1996). In fact, several past studies have shown the importance of syllable awareness in English reading among Chinese ESL children (e.g., Chow, McBride-Chang, & Burgess, 2005; McBride-Chang et al., 2004).

Mastery of alphabetic principles has been suggested as a key route of word reading of alphabetic orthographies by beginning readers (Ehri, 1991). To engage in such a process, children need to develop phoneme awareness and knowledge in letter-sound correspondences. Auditory-driven phonological awareness programs without teaching letter-sound correspondences, like the one being examined in this study, are effective in promoting the reading development of L1 kindergarteners (Lundberg, Frost, & Petersen, 1988; Cunningham, 1990; Lie, 1991). The findings of this study have shown that children made significant gains in phoneme awareness and that the enhanced phoneme awareness accounted for additional unique variance of improvement in word reading. This suggests that, once children acquire phoneme awareness, even without explicit instruction in letter-sound correspondences, they change the reading strategy and readily apply the phoneme awareness to aid word recognition. This pattern is remarkably similar to that shown by monolingual children who learn English as a L1 (Trieman, Sotak, & Bowman, 2001). The findings echo previous studies that show that the developmental trajectory of ESL learners in reading acquisition is similar to native speaking children (Jongejan, Verhoeven, & Siegel, 2007; Lesaux & Siegel, 2003). It has been suggested that the ability to segment words consciously into constituent phonemes and blend phonemes into words, together with sufficient letter knowledge, predicts beginning English reading development (Foorman et al., 2003). We have shown that instruction in phoneme segmentation, which is primarily auditory in nature, is beneficial to early English reading development in Chinese ESL children who have very weak initial phoneme awareness. Phoneme blending is relatively difficult for young children in Hong Kong. Such research informs us more about the characteristics of effective reading instruction for young ESL learners.

It is worth noting that the existing English language curriculum emphasizes the teaching of letter names and participants in this research generally had high levels of letter name knowledge but poor phonological awareness, in particular phoneme awareness. This is in contrast to the view that knowledge of letters alone is a sufficient condition for phoneme awareness to develop (Castle & Coltheart, 2004; Mann & Wimmer, 2002). Rather, the current findings support that both phonological awareness instruction and the development of letter knowledge promote reading ability (Byrne, 1998; Caravolas, Hulme, & Snowling, 2001).

In learning to read, Perfetti and colleagues (Perfetti, 2003; Perfetti & Liu, 2005) proposed that children first need to acquire the language elements that are encoded in the writing system. This is referred to as the general mapping principle. In English, it means that children must understand that each letter represents a distinct sound, i.e. phonological awareness. Then, the details of the letter-sound relationships, the mapping details, need to be acquired gradually. The existing reading instruction for Hong Kong young children does not usually address the foundation skills for learning to read (the acquisition of general mapping principles). The present research has shown that the acquisition of this general mapping principle is important for the beginning reading development of L2 children whose L1 is non-alphabetic, because the prior experience of L1 cannot facilitate the learning of such a principle. For Chinese children, both the general and detailed mapping principles need to be taught explicitly in reading instruction.

Interestingly, for children who have not undergone any phonological awareness instruction, oral language skills, but not phonological awareness, predicted word reading. The reverse pattern was characteristic of children in the instructional group (see Table 3). We suggest that when children are not taught phonological awareness explicitly, under the framework of dual route model of reading (Coltheart, Curtis, Atkins, & Haller, 1993), they rely more on the lexical route in reading and thus oral language skills are relatively important. When children have acquired a certain level of phonological awareness and are able consciously to segment words into smaller phonological units, the sublexical route is used in the reading process.

The significant improvement of expressive vocabulary as shown in the current study is another encouraging finding because vocabulary learning has been well acknowledged as difficult and lengthy (Beck & McKeown, 1991). Consistent with past studies of ESL children (e.g., Gottardo et al., 2008), the present research has shown that expressive vocabulary is related closely to English word reading, as suggested by the lexical restructuring hypothesis. This highlights the role of oral language skills in designing effective reading instruction for Chinese ESL children and suggests that instructional strategy should incorporate elements of these skills, in particular, expressive vocabulary. Our data showed that only expressive vocabulary but not receptive vocabulary significantly improved among children in the instructional group. A possible explanation is that the receptive vocabulary measure employed in this study was a Western measure that may include items that are unfamiliar or culturally meaningless to Hong Kong students. Consequently, task sensitivity to individual differences might be compromised. On the other hand, the picture naming task, in which students were asked to name objects or animals, was very familiar to the participants, as they are often asked to do so in English lessons. The testing procedures of the picture naming task was also similar to the learning activities in the instructional program which might indirectly promoted the performance on this task at Time 2.

The program used in this study, being both explicit and systematic, showed instructional elements that have been well documented to be effective in promoting reading success in both L1 and L2 learners (Foorman & Torgesen, 2001). Concrete and teacher-friendly curriculum materials have been noted as important for program success in promoting reading performance (Fuchs et al., 2001). These critical elements are conditions for children of varying levels of skill and background to benefit from phonological awareness programs. The encouraging findings of this study support the design and implementation of this program.

### Educational implications

The positive effects of phonological awareness instruction on beginning reading point to the need to teach analytical skills in phonology to help Chinese ESL learners to acquire L2 reading skills. Explicit and direct instruction in helping children to acquire the alphabetic principle has been demonstrated to be a key in learning to read in English (Rayner, Foorman, Perfetti, Pesetsky, & Seidenberg, 2001). However, the teaching of English in many Chinese communities still has a heavy emphasis on the whole word approach. Educators in the field may consider how to incorporate phonological elements into early English learning in preschool settings.

The importance of oral language proficiency, as demonstrated in the present study, suggests that mastery of L2 oral skills along with phonological awareness may be beneficial for Hong Kong young children who have high literacy demands in both Chinese and English. In many Hong Kong preschool classrooms, the teaching of English emphasizes writing exercises and children in general have little exposure to oral language (limited to 40–60 min per week provided by NET in the English lesson times). ESL children have been reported to be lagging behind their L1 counterparts in vocabulary development (Jean & Geva, 2009). They are also less likely to learn vocabulary via incidental learning or text reading (Proctor, Carlo, August, & Snow, 2005), with explicit instruction needed (August, Carlo, Dressler, & Snow, 2005).

### Conclusion

This study has extended the current literature by showing that phonological awareness instruction delivered in classroom settings is beneficial not only to phonological awareness but also, to a lesser extent, to the beginning reading and spelling skills of young Chinese ESL children. Our findings suggest that young Chinese ESL children, as early as kindergarten, are similar to other ESL children with different L1 in responding to phonological awareness instruction despite great differences in L1 and educational experiences. However, it has been argued that phonological awareness is not the only factor in the process of learning to read (Bus & van IJzendoorn, 1999) and oral language skills deserve more research attention in reading instruction studies (Duff et al., 2008). It is proposed that integrated phonological awareness plus language learning are important in designing effective instruction for early reading for Chinese ESL children.

## Footnotes

1 Considering the poor pronunciation of Hong Kong Chinese kindergarteners, scoring criteria were developed to ensure that the comparison group would not be disadvantaged because they have less exposure to phoneme instruction. If the pronounced English word can be recognized by the experimenters as the targeted word, it is regarded as a correct response. This criterion was endorsed for both groups of children and for both time points of assessment.

## References

[CR1] Adams M (1990). Beginning to read: Thinking and learning about print.

[CR64] Anthony, J. L., Lonigan, C. J., Driscoll, K., Phillips, B. M., & Burgess, S. R. (2003). Phonological sensitivity: A quasi-parallel progression of word structure units and cognitive operations. *Reading Research Quarterly, 38*(4), 470–487.

[CR2] August D, Carlo M, Dressler C, Snow C (2005). The critical role of vocabulary development for English language learners. Learning Disabilities Research & Practice.

[CR3] August, D., & Hakuta, K. (1997). *Improving schooling for language*-*minority students: A research agenda* (Committee on Developing a Research Agenda on the Education of Limited English Proficient and Bilingual Students—Board on Children, Youth and Families). Washington, DC: National Academy Press.

[CR4] Ayres LR, Weaver C (1998). Phonological awareness training of kindergarten children: Three treatments and their effects. Reconsidering a balanced approach to reading.

[CR5] Beck I, McKeown M, Barr R (1991). Conditions of vocabulary acquisition. Handbook of reading research.

[CR6] Bennett L, Ottley P (2000). Launch into reading success through phonological awareness training.

[CR7] Bernhardt EB, Kamil ML (1995). Interpreting relationships between L1 and L2 reading: Consolidating the linguistic threshold and the linguistic interdependence hypotheses. Applied Psycholinguistics.

[CR8] Blachman BA, Blachman BA (1997). Early intervention and phonological awareness: A cautionary tale. Foundations of reading acquisition and dyslexia: Implications for early intervention.

[CR9] Bradley L, Bryant PE (1983). Categorizing sounds and learning to read: A causal connection. Nature.

[CR10] Bus AG, van IJzendoorn MH (1999). Phonological awareness and early reading: A meta-analysis of experimental training studies. Journal of Educational Psychology.

[CR11] Byrne B (1998). The foundation of literacy: The child’s acquisition of the alphabetic principle.

[CR12] Caravolas M, Hulme C, Snowling M (2001). The foundation of spelling ability: Evidence from a 3-year longitudinal study. Journal of Memory and Language.

[CR13] Castle A, Coltheart M (2004). Is there a causal link from phonological awareness to success in learning to read?. Cognition.

[CR14] Cheung H, Chen H-C, Yip-Lai C, Wong OC, Hills M (2001). The development of phonological awareness: Effects of spoken language experience and orthography. Cognition.

[CR15] Cheung H, Chung KKH, Wong SWL, McBride-Chang C, Penney TB, Ho CSH (2010). Speech perception, metalinguistic awareness, reading, and vocabulary in Chinese–English bilingual children. Journal of Educational Psychology.

[CR16] Chiappe P, Siegel LS, Wade-Woolley L (2002). Linguistic diversity and the development of reading skills: A longitudinal study. Scientific Studies of Reading.

[CR65] Chow, B. W.-Y., McBride-Chang, C., & Burgess, S. (2005). Phonological processing skills and early reading abilities in Hong Kong Chinese kindergarteners learning to read english as a second language.* Journal of Educational Psychology, 97*(1), 81–87.

[CR17] Coltheart M, Curtis B, Atkins P, Haller M (1993). Models of reading aloud: Dual-route and parallel-distributed-processing approaches. Psychological Review.

[CR18] Cunningham AE (1990). Explicit versus implicit instruction in phonemic awareness. Journal of Experimental Child Psychology.

[CR19] Duff FJ, Fieldsend E, Bowyer-Crane C, Hulme C, Smith G, Gibbs S, Snowling M (2008). Reading with vocabulary intervention: Evaluation of an instruction for children with poor response to reading intervention. Journal of Research in Reading.

[CR20] Dunn LM, Dunn LM (1981). Peabody picture vocabulary test-revised.

[CR21] Ehri LC, Barr R, Kamil ML, Mosenthal P, Pearson PD (1991). Development of the ability to read words. Handbook of reading research.

[CR22] Ehri LC, Nunes SR, Willows DM, Schuster BV, Yaghoub-Zadeh Z, Shanahan T (2001). Phonemic awareness instruction helps children learn to read: Evidence from the National Reading Panel’s meta-analysis. Reading Research Quarterly.

[CR23] Foorman BR, Chen DT, Carlson LM, Francis DJ, Fletcher JM (2003). The necessity of the alphabetic principle to phonemic awareness instruction. Reading and Writing: An Interdisciplinary Journal.

[CR24] Foorman BR, Torgesen J (2001). Critical elements of classroom and small-group instruction promote reading success in all children. Learning Disabilities Research & Practice. Special Issue: Emergent and Early Literacy: Current Status and Research Directions.

[CR66] Fuchs, D., Fuchs, L. S., Thompson, A., Otaiba, S. A., Yen, L., Yang, N. J., Brown, M., O’Connor, R. E. (2001). Is reading important in reading-readiness programs? A randomized field trial with teacher as program implementers. *Journal of Educational Psychology, 93*(2), 251–267.

[CR25] Ganschow L, Sparks R (1995). Effects of direct instruction in Spanish phonology on the native-language skills and foreign-language aptitude of at-risk foreign-language learners. Journal of Learning Disabilities.

[CR26] Gerber M, Jimenez T, Leafstedt J, Villaruz J, Richards C, English J (2004). English reading effects of small-group intensive intervention in Spanish for K-l English learners. Learning Disabilities Research & Practice.

[CR27] Gottardo A, Collins P, Baciu I, Gebotys R (2008). Predictors of grade 2 word reading and vocabulary learning from grade 1 variables in Spanish-speaking children: Similarities and differences. Learning Disabilities Research & Practice.

[CR28] Gottardo A, Yan B, Siegel LS, Wade-Woolley L (2001). Factors related to English reading performance in children with Chinese as a first language: More evidence of cross-language transfer of phonological processing. Journal of Educational Psychology.

[CR29] Hanley JR, Tzeng O, Huang HS, Harris M (1999). Learning to read Chinese. Learning to read and write: A cross-linguistic perspective. Cambridge studies in cognitive and perceptual development.

[CR30] Holm A, Dodd B (1996). The effect of first written language on the acquisition of English literacy. Cognition.

[CR31] Huang HS, Hanley JR (1995). Phonological awareness and visual skills in learning to read Chinese and English. Cognition.

[CR32] Jean M, Geva E (2009). The development of vocabulary in English as a second language children and its role in predicting word recognition ability. Applied Psycholinguistics.

[CR33] Jongejan W, Verhoeven L, Siegel LS (2007). Predictors of reading and spelling abilities in first- and second-language learners. Journal of Educational Psychology.

[CR34] Keung YC, Ho CSH (2009). Transfer of reading related cognitive skills in learning to read Chinese (L1) and English (L2) among Chinese elementary school children. Contemporary Educational Psychology.

[CR35] Leafstedt JM, Richards CR, Gerber MM (2004). Effectiveness of explicit phonological-awareness instruction for at-risk English learners. Learning Disabilities Research & Practice.

[CR36] Learning Disabilities Association of Alberta (2009). Reading readiness screening tool.

[CR37] Lesaux NK, Siegel LS (2003). The development of reading in children who speak English as a second language. Developmental Psychology.

[CR38] Lie A (1991). Effects of a training program for stimulating skills in word analysis in first-grade children. Reading Research Quarterly.

[CR39] Lindsey KA, Manis FR, Bailey CE (2003). Prediction of first-grade reading in Spanish-speaking English-language learners. Journal of Educational Psychology.

[CR40] Luk G, Bialystok E (2008). Common and distinct cognitive bases for reading in English–Cantonese bilinguals. Applied Psycholinguistics.

[CR41] Lundberg I, Frost J, Petersen O-P (1988). Effects of an extensive program for stimulating phonological awareness in preschool children. Reading Research Quarterly.

[CR42] Mann V, Wimmer H (2002). Phoneme awareness and pathways into literacy. A comparison of German and American children. Reading and Writing: An Interdisciplinary Journal.

[CR43] McBride-Chang C, Bialystok E, Chong KKY, Li Y (2004). Levels of phonological awareness in three cultures. Journal of Experimental Child Psychology.

[CR44] McBride-Chang C, Ho CS-H (2005). Predictors of beginning reading in Chinese and English: A 2-year longitudinal study of Chinese kindergartners. Scientific Studies of Reading.

[CR45] McBride-Chang C, Kail RV (2002). Cross-cultural similarities in the predictors of reading acquisition. Child Development.

[CR46] McBride-Chang C, Treiman R (2003). Hong Kong Chinese kindergartners learn to read English analytically. Psychological Science.

[CR47] Meador D, Flege JE, MacKay IR (2000). Factors affecting the recognition of words in a second language. Bilingualism: Language and Cognition.

[CR48] Metsala JL, Walley AC, Metsala JL (1998). Spoken vocabulary growth and the segmental restructuring of lexical representations: Precursors to phonemic awareness and early reading ability. Word recognition in beginning literacy.

[CR49] Muter V, Hulme C, Snowling M, Taylor S (1997). Segmentation, not rhyming, predicts early progress in learning to read. Journal of Experimental Child Psychology.

[CR50] Muter V, Snowling M (1998). Concurrent and longitudinal predictors of reading: The role of metalinguistic and short-term memory skills. Reading Research Quarterly.

[CR51] Nation K, Snowling MJ (2004). Beyond phonological skills: Broader language skills contribute to the development of reading. Journal of Research in Reading.

[CR52] National Reading Panel. (2000). *Teaching children to read: An evidence-based assessment of scientific research literature on reading and its implications for reading instruction* (NIH Publication No. 00-4769).

[CR53] Perfetti CA (2003). The universal grammar of reading. Scientific Studies of Reading.

[CR54] Perfetti CA, Liu Y (2005). Orthography to phonology and meaning: Comparisons across and within writing systems. Reading and Writing: An Interdisciplinary Journal.

[CR55] Proctor PC, Carlo M, August D, Snow C (2005). Native Spanish-speaking children reading in English: Toward a model of comprehension. Journal of Learning Disabilities.

[CR56] Raven JC, Court JH, Raven R (1976). Manual for Raven’s progressive matrices and vocabulary scales. Section III: Standard progressive matrices.

[CR57] Rayner K, Foorman BR, Perfetti CA, Pesetsky D, Seidenberg MS (2001). How psychological science informs the teaching of reading. Psychological Science in the Public Interest.

[CR58] Snow CE, Burns MS, Griffin P, Snow CE, Burns MS, Griffin P (1999). Preventing reading difficulties in young children. Reading research: Anthology: The why of reading instruction.

[CR59] Torgesen JK, Wagner RK, Rashotte CA (1997). Prevention and remediation of severe reading disabilities: Keeping the end in mind. Scientific Studies of Reading. Special Issue: Components of Effective Reading Instruction.

[CR60] Trieman R, Sotak L, Bowman M (2001). The roles of letter names and letter sounds in connecting print and speech. Memory and Cognition.

[CR61] Wagner RK, Torgesen JK, Rashotte CA (1994). Development of reading-related phonological processing abilities: New evidence of bidirectional causality from a latent variable longitudinal study. Developmental Psychology.

[CR62] Whitehurst GJ, Lonigan CJ (1998). Child development and emergent literacy. Child Development.

[CR63] Ziegler JC, Goswami U (2005). Reading acquisition, developmental dyslexia, and skilled reading across languages: A psycholinguistic grain size theory. Psychological Bulletin.

